# Contribution of cognitive and bodily navigation cues to egocentric and allocentric spatial memory in hallucinations due to Parkinson's disease: A case report

**DOI:** 10.3389/fnbeh.2022.992498

**Published:** 2022-10-13

**Authors:** Cosimo Tuena, Giuseppe Riva, Immacolata Murru, Luca Campana, Karine M. Goulene, Elisa Pedroli, Marco Stramba-Badiale

**Affiliations:** ^1^Applied Technology for Neuro-Psychology Lab, IRCCS Istituto Auxologico Italiano, Milan, Italy; ^2^Humane Technology Lab, Università Cattolica del Sacro Cuore, Milan, Italy; ^3^Department of Geriatrics and Cardiovascular Medicine, IRCCS Istituto Auxologico Italiano, Milan, Italy; ^4^Department of Neurology and Laboratory of Neuroscience, IRCCS Istituto Auxologico Italiano, Milan, Italy; ^5^Faculty of Psychology, Università eCampus, Novedrate, Italy

**Keywords:** embodiment, virtual reality, spatial navigation, Lewy bodies, psychosis

## Abstract

Parkinson's disease (PD) manifestations can include visual hallucinations and illusions. Recent findings suggest that the coherent integration of bodily information within an egocentric representation could play a crucial role in these phenomena. Egocentric processing is a key aspect of spatial navigation and is supported by the striatum. Due to the deterioration of the striatal and motor systems, PD mainly impairs the egocentric rather than the allocentric spatial frame of reference. However, it is still unclear the interplay between spatial cognition and PD hallucinations and how different navigation mechanisms can influence such spatial frames of reference. We report the case of A.A., a patient that suffers from PD with frequent episodes of visual hallucinations and illusions. We used a virtual reality (VR) navigation task to assess egocentric and allocentric spatial memory under five navigation conditions (passive, immersive, map, path decision, and attentive cues) in A.A. and a PD control group without psychosis. In general, A.A. exhibited a statistically significant classical dissociation between the egocentric and allocentric performance with a greater deficit for the former. In particular, the dissociation was statistically significant in the “passive” and “attentive cues” conditions. Interestingly in the “immersive” condition, the dissociation was not significant and, in contrast to the other conditions, trends showed better performance for egocentric than allocentric memory. Within the theories of embodiment, we suggest that body-based information, as assessed with VR navigation tasks, could play an important role in PD hallucinations. In addition, the possible neural underpinnings and the usefulness of VR are discussed.

## Introduction

Parkinson's disease (PD) psychosis refers to a range of delusions, hallucinations, and illusions that can occur throughout the disease (Albani et al., 2015; Ffytche et al., 2017). Historically, hallucinations were thought to be related to dopaminergic therapy, however, new studies show that they could be intrinsically linked to neurodegeneration due to PD (i.e., Lewy bodies) (Williams and Lees, 2005; Diederich et al., 2009; Bertram and Williams, 2012; Glass et al., 2012; Muller et al., 2014).

Different theories have been proposed to explain how and why visual hallucination occurs in PD. These include the perception and attention deficit model, in which impairments in top-down (attention) and bottom-up (perceptual) processing lead to the occurrence of hallucination (Collerton et al., 2005), the reality monitoring deficit model (i.e., inability to judge the source of a perception; Barnes et al., 2003), the dream imagery intrusion model (i.e., intrusion of endogenous imagery produced during dreaming; Arnulf et al., 2000), the altered gating of external and internal imagery model (Diederich et al., 2005), or the brain networks hypothesis (Onofrj et al., 2013). In particular, the default mode network comprises medial frontal, temporal, and parietal cortices activated during rest and episodic memory, social cognition, envisioning the future, and spatial navigation (Buckner and Carroll, 2007; Buckner et al., 2008). Onofrj and colleagues (Franciotti et al., 2013; Onofrj et al., 2013) suggest that the lack of hypoactivation in this network and particularly the posterior cingulate cortex might play a crucial role in the attention brain networks and the formation of visual hallucinations.

More recently, Bernasconi et al. (2021) studied the mechanisms behind hallucinations in patients with PD. The researchers identified a subgroup of patients who were more sensitive to experimentally robot-induced presence hallucination and found that frontotemporal connectivity, which was linked to hallucinations in healthy people, was altered in patients with PD who had clinically reported hallucinations. Previously, Arzy et al. (2006) showed that the stimulation of the temporoparietal junction in an epileptic patient altered the multisensory integration of bodily information and induced a presence illusion. These findings support the importance of the processing of sensorimotor information within a coherent bodily representation in the development of visual hallucinations/illusions as suggested also by recent theories (Riva, 2018; Corlett et al., 2019). It is possible that bottom-up egocentric (body-based) sensorimotor processing and abnormal top-down perceptual expectations in psychosis make patients more susceptible to illusions that concern the integration of body-based information with higher-order representations (Corlett et al., 2019).

Egocentric processing has been consistently investigated within the spatial cognition domain. The egocentric spatial frame of reference is defined as a representation of the space based on one's body and perspective (Burgess, 2008). Conversely, in the allocentric frame of reference, space is represented in a cognitive map independently of one's body (Burgess, 2008). Spatial navigation relies on these two frames of reference to remember items' locations using, respectively, landmarks (supported by the striatum and parietal cortex) and boundaries (mainly by the right hippocampus) of the space (Chersi and Burgess, 2015). However, spatial navigation, in addition to vision, requires the active recruitment of bodily information (e.g., motor commands, proprioception, vestibular system) and cognitive processes (e.g., spatial attention, spatial operations, and route decision-making) (Chrastil and Warren, 2012). How these cues influence egocentric and allocentric frames of reference is still a matter of debate (Chrastil and Warren, 2012; Huffman and Ekstrom, 2021; Steel et al., 2021). In particular, some authors (e.g., Huffman and Ekstrom, 2021) suggest that egocentric spatial memory is more affected by body-based information or peri-personal space tasks.

Different studies showed that egocentric spatial cognition is impaired in PD due to the degeneration of the basal ganglia and motor system (Humphries et al., 2016; Thurm et al., 2016; Kuehn et al., 2017; Fernandez-Baizan et al., 2020). In contrast, studies that evaluate spatial cognition in PD patients with visual hallucinations are still preliminary. Space perception was found to be impaired in PD with visual hallucinations compared to PD without hallucinations (Koerts et al., 2010). In another study (Barnes and Boubert, 2011), PD patients with hallucinations were found to be impaired on a spatial location memory task compared to the PD group without hallucinations and healthy controls. However, it is still unclear what is the impact of hallucinations due to PD on the spatial frames of reference depending on different cognitive (i.e., attention, planning, and spatial reasoning) and bodily (i.e., idiothetic information and visual only) involvement.

Interestingly, virtual reality (VR) is a powerful tool that has been used consistently in the literature to tap egocentric and allocentric spatial memory and navigation in neurological conditions thanks to its potential to provide multisensory experiences as close as real-world navigation, especially when used with an immersive apparatus (Tuena et al., 2021a,b).

Case reports with innovative methods can provide valuable information (e.g., Callesen et al., 2013; Hartevelt et al., 2015), here, we wanted to explore if the egocentric and allocentric frames of reference are differently affected in a patient with PD and recurrent visual hallucinations with different VR navigation interfaces that involve cognitive and bodily information. To pursue this aim, we applied CARE (CAse-REport) guidelines (Riley et al., 2017) and the neuropsychological dissociation criteria and statistical tests (Crawford and Garthwaite, 2005) proposed by McIntosh (2018) to assess the difference between the egocentric and allocentric performance.

Our study could shed new light on how egocentric and allocentric processing is modified depending on the involvement of specific cognitive and bodily information.

## Methods

### Participants

A.A. is a 75-year-old man affected by PD (Queen Square Brain Bank criteria; Berardelli et al., 2013) since 2018. A.A. presents a left predominant akinetic-rigid PD with rare tremor episodes, which manifested early in the disease, and infrequent episodes of freezing of gait. He requires no or minimal assistance for daily life activities (ADL 5/6, IADL 8/8). During medical examinations carried out in 2021, mild symptoms of depression, mild hypomimia, and hypophonia were observed. He also reported ageusia and anomia. At the time of the diagnosis (2018), an MRI examination showed mild degenerative (bilateral frontotemporal and Sylvian sulci) atrophy and absence of acute ischemic lesions. Despite the observed atrophy, neuropsychological tests were within the normal range at that time. Dopaminergic therapy was started in August 2018 with symptomatology improvement. The idiopathic PD diagnosis was improved in 2019 through the dopamine transporter (DaT) SPECT scan technique that showed a severe reduction in the density of presynaptic DaT in both the striates, more affected on the right side (same pattern for the caudate, putamen, and the putamen/caudate ratio). The scores of DaT scan were the followings: global striatum (0.48, 0.55; right and left respectively; normative values 1.5–2.9), caudate (0.67, 0.79; normative values 1.7–3.3), putamen (0.29, 0.39; normative values 1.4–3.1), and putamen/caudate ratio (0.44, 0.49; normative values >0.7). In June 2021 during the physiatrist visit, he reported balance worsening in the previous six months and subjective memory complaint with episodes of anomia. Left tactile hypoesthesia, presence of retropulsion, *marche à petit pas*, and positive Romberg test were observed; dysdiadochokinesia and buccofacial praxis were preserved.

During this period dopaminergic therapy was set during a neurological visit in January 2021 with Madopar 200/50 mg ¼ four times during the day (hours 6–11.30–17.30–23). A neuropsychological evaluation carried out in June 2021 revealed a mild cognitive impairment (Litvan et al., 2012). In particular, selective attention was found to be impaired (2 SD below the population's mean; Goldman et al., 2013); however borderline deficits were also found for phonemic fluency, auditory-verbal short-term memory, visuospatial short-term memory, and immediate auditory-verbal long-term memory tests. Global cognition (Addenbrooke's cognitive examination revised; ACE-R = 86.23; Pigliautile et al., 2015), reasoning, delayed visual and auditory-verbal memory, constructional apraxia, and executive functions tests were within the normal range. Physiotherapy and cognitive stimulation were started after these evaluations in June 2021. In October 2021 during the neurological follow-up visit, the dopaminergic therapy was continued with Madopar 200/50 mg ¼ for four times (hour 8–12–16–20). In December 2021, he was enrolled in a VR project (ANTaging) that aimed at assessing and rehabilitating spatial memory in patients with mild cognitive impairment with VR. During this period, he reported a worsening of visual hallucinations to the experimenter.

During the clinical examination (April 2022) of visual hallucinations, the patient reported having simple and complex visual hallucinations for more than 2 years, surly before the COVID-19 pandemic, but after the diagnosis of PD. Hallucinations are experienced almost every day during the day and particularly in the evening. Hence, the criteria for the PD psychosis diagnosis were met (Diederich et al., 2009): at least one among illusions, sense of presence, hallucinations, and/or delusions; symptoms occurred after the diagnosis of PD; symptoms are recurrent for at least a month; not due to other psychiatric or medical conditions. In particular, he reported seeing bugs moving close to him, though not infesting him, almost every day. Sense of a presence is reported less frequently but weekly and usually it consists of a dark shadow on the side moving from one side to the other in front of him. He also reported what he called ‘lightnings’ moving from the periphery of the visual field toward the center that now are less frequent. He feels controlled by his wife, who is naturally concerned about his condition. He reported no auditory/olfactory hallucinations, palinparousia, pareidolia, or hallucinations during the VR sessions. His insight is preserved, and he is aware that these phenomena are unreal, and uses this as a psychological coping strategy. In May 2022, MRI examination was repeated and did not reveal any clinically significant changes from the one in 2018.

Hence, the inclusion criteria for the case report were: PD diagnosis (Berardelli et al., 2013); presence of mild cognitive impairment (Litvan et al., 2012); PD drug therapy (i.e., levodopa); absence of ischemic lesions to exclude pure vascular parkinsonism (Vizcarra et al., 2015); presence of visual hallucinations and illusions.

In addition to A.A., we included a control group of five (*M*_age_ = 79.6, SD_age_ = 2.06; *M*_edu._ = 13.2, SD_edu_ = 3.87; four males; *M*_ACE − R_ = 80.09, SD_ACE − R_ = 5.65; *M*_ADL_ = 5.2, SD_ADL_ = 1.6) PD patients without psychosis that were enrolled in the same period of A.A. in the ANTaging project. For the control group, the inclusion criteria were: PD diagnosis (Berardelli et al., 2013); presence of mild cognitive impairment (Litvan et al., 2012); PD drug therapy (i.e., levodopa); absence of ischemic lesions to exclude pure vascular parkinsonism (Vizcarra et al., 2015); absence of hallucinations and illusions of any kind. This group was also balanced for motor symptoms asymmetry onset (three right side, two left side), years from the diagnosis (*M* = 4.1, SD = 3.85), levodopa daily dose (*M* = 292.5, SD = 190.31), and all the patients had a predominant akinetic-rigid phenotype.

Exclusion criteria for the case report and the control group were: PD dementia; presence of physical and/or functional deficits that could hamper the use of VR; visual field deficits; recurrent vertigo; acute stroke; other severe concomitants neurological and/or psychiatric diseases; history of traumatic brain injury with loss of consciousness. Tables 1, 2 show demographics and neuropsychological and psychosis assessments.

**Table 1:** Summary of demographics and clinical variables.

	**Control group**	**A.A**.	***p*-value**
	**(N = 5)**		
Age	79.6 (2.06)	75	NS
Education	13.2 (3.87)	8	NS
ACE-R Total	80.09 (5.65)	86.23	NS
ACE-R (AO)	17.8 (0.4)	17	NS
ACE-R (M)	20.8 (3.87)	21	NS
ACE-R (F)	8.4 (0.49)	7	NS
ACE-R (L)	23.8 (2.04)	23	NS
ACE-R (VS)	14.2 (1.17)	16	NS
CBT	4.42 (0.19)	4.5	NS
CSS	10.78 (6.68)	6.36	NS
GDS	6.6 (1.85)	6	NS
ADL	5.2 (1.6)	5	NS
LDD (mg)	292.50 (190.31)	200	NS
Disease duration (yrs)	4.1 (3.85)	4	NS

Mean and SD are shown for the control group.

ACE-R, Addenbrooke's cognitive examination revised; AO, attention and orientation; M, memory; F, phonological and semantic fluency; L, language; VS, visuospatial; LDD, levodopa daily dose; CBT, Corsi block-tapping test; CSS, Corsi supra-span test; GDS, geriatric depression scale; ADL: activities of daily living.

**Table 2:** Psychosis evaluation.

	***z*-Score**
NMSS (perceptual problems/hallucinations)	1.67
PS (frequency visual)	0.89
PS (frequency olfactory)	−0.45
PS (frequency auditory)	−0.77
PS (frequency presence)	−0.04
PS (delusion assessment)	−0.69
PS (duration of psychosis)	−0.73
PS (absence of insight)	−1.06
PS (threatening)	−0.57
PS (interaction)	−0.87
PS (family concern)	0.07
PS total	−0.67

NMSS z-score refers to the Italian normative data, whereas PS sub-scores and total z-score refer to normative data of non-Italian PD patients with psychosis. Positive z-score represents more severe symptoms.

NMSS, non-motor symptoms scale; PS, psychosis scale.

This case report was approved by the Ethical Committee of Istituto Auxologico Italiano and patients enrolled in the study signed the consent form to retrospectively use the data collected in the ANTaging project for this new research.

### Egocentric and allocentric task

To test egocentric and allocentric spatial memory in our participants, we used a VR landmark-based navigation task (Guderian et al., 2015). In the encoding phase, participants were asked to collect and memorize the position of four objects in a circular arena (the diameter of the arena was 50 virtual meters). Object locations could be remembered using the boundaries of the arena (i.e., wall), an intra-arena landmark (i.e., obelisk), and distal cues (i.e., mountain range, fixed clouds). Objects were randomly presented, and each object was collected four times in random order. Items were presented one at the time. To see the following object, the participants had to go to the exact location of the item. Once over it, the object disappeared, and the patient had to find the next one.

During the immediate recall phase, participants had to remember and go to the exact location where the item was previously collected and press the spacebar to respond. Then the following object was shown. In random order, either the wall or the obelisk was removed. This forced the use of allocentric (i.e., wall) or egocentric (i.e., obelisk) spatial memory recall. Each object was tested four times with the egocentric and allocentric spatial frame for a total of 16 trials (eight trials for each allocentric and egocentric recall condition). The response variable was the distance error for each object trial at recall (distance in virtual meters of the recalled position from the actual location). The greatest error possible is 49 virtual meters.

### Procedure

Participants were tested on the task described above with different interfaces according to the active and passive navigation characteristics (Chrastil and Warren, 2012). Participants' egocentric and allocentric spatial memory was assessed with five navigational interfaces (passive, immersive, map, path decision, and attentive cues) in two experimental sessions (1 h 15 min for each session; 2–3 days distance).

The “passive” and “immersive” interfaces were tested in the *sensorimotor session*, while the remaining three were used in the *cognitive session*. The two blocks were counterbalanced among participants, and the interfaces were randomized within each session. Four objects were chosen in each condition by the VR spatial task software from a random list of eight. Each random pick balanced the living and non-living categories of items.

Participants in the “immersive” condition were immersed in the environment with a 3D visor (Oculus Rift S) and could move with the 3dRudder (https://www.3drudder.com/). In addition, in the learning phase they used directional cues (a line to follow to reach the item) but no interactive map (see below for description) or attentional cues (see below for description). This is done to separate the bodily component (i.e., motor commands, proprioception, and vestibular system) from the rest of the active processes (i.e., inhibiting route decision-making, spatial manipulation, and spatial attention). The same VR apparatus is used in the recall phase, but no directional cues, maps, or attentive cues are provided. Participants in the “passive” condition simply observed the experimenter's navigation on the PC screen (no map, directional and attentive cues). This is done to isolate the visual system as if the participant was seated in a car like a passenger.

To reduce the involvement of bodily information (especially proprioception and vestibular information; Taube et al., 2013) in the cognitive session, 2D VR (PC screen, keyboard keys, and mouse) was used during the encoding and recall phases. The participant in the “map” condition navigated using an interactive map (a map that rotates depending on direction and gives cardinal points, landmarks, and items locations) and directional cues but no attentional cues (i.e., inhibiting route decision-making, bodily information, and spatial attention). During the recall phase, all the cues were not provided, and the patients used the 2D VR set to find the items' locations. Before the learning phase in the “attentive cues” condition, the participant was at the center of the arena with the obelisk, wall, and distal cues, and he/she was asked to look around with the mouse, discover six orange markers, and loudly say the number above each marker. Markers were placed one on the obelisk, one on the mountain range, and four on the top of the wall (circularly equidistant). This circumstance necessitated the employment of attentive resources to the spatial layout and environment before navigation. The experimenter made all the markers vanish once they were correctly found. Then the encoding phase started, and the patient was given directional clues but no map during the learning process (i.e., inhibiting route decision-making, bodily information, and spatial manipulation). Attentional and directional cues were not presented during recollection and the patients used the 2D VR set to find the item locations. Finally, directional cues were removed from the “path decision” interface at encoding and neither the map nor attentive cues were provided (i.e., inhibiting spatial attention, bodily information, and spatial manipulation). In this sense, the participant was free to choose where to go. There was no cue during the recall phase and the patients used the 2D VR set to find the item locations.

Patients were motivated by instructions provided in the form of a short story in which they were asked to assist a little girl in collecting, remembering, and replacing the items in the arena. Each story was modified according to the navigation interface. These instructions were read before the encoding (find and encode objects' locations) and retrieval phase (put back the objects where collected). Neuropsychological tests were carried out at the end of the sensorimotor block so that each block consisted of the same amount of cognitive load (three cognitive tasks for each block). Each navigation condition (encoding plus immediate recall) was separated by a pause of 5 min where the patients filled out a questionnaire (GDS, ADL, or IADL). Before the start of the sensorimotor or cognitive block depending on the counterbalanced order, patients read and signed the consent forms to participate in the ANTaging protocol. See Supplementary material 1 for a video demo of the task with only one object location.

### Statistical analyses

Statistical analyses were carried out using R (v. 3.6.3) (R Core Team, 2013). To test for a classical dissociation between egocentric and allocentric performance, we used the criteria proposed by McIntosh (2018). The criteria suggest the use of the revised standardized difference test (RSDT) (Crawford and Garthwaite, 2005) and effect size for the test of difference (*Z*_DCC_) (Crawford et al., 2010) to describe respectively the presence and strength of the dissociation. McIntosh criteria combined with RSDT allow to retain great control over Type I error also with small group. The required control group sample size for RSDT and the McIntosh criteria is at least *N* = 5. In addition, the modified (two-tailed) Crawford et al. (1998) *t*-test was used to compare neuropsychological tests performances and clinical variables of A.A. with the control group (see Table 1). *Singcar* package was used to perform Crawford's tests. Spearman's rank correlation was used to assess the association between the dependent variable (i.e., error) and testing trials in the global egocentric and allocentric performance in A.A. and the control group.

We tested egocentric and allocentric memory performance for global (regardless of the navigation interfaces) and each navigation condition. In the former analysis, for each participant, an average error performance was computed within trials and across the navigation interfaces. For A.A. a total of 40 egocentric trials and 40 allocentric trials were averaged across the five navigational interfaces. One patient in the control group could not complete the “immersive” navigation due to motion sickness discomfort, hence in the control group 192 egocentric and 192 allocentric spatial frame trials were averaged. Similarly, to analyze the impact of each navigation interface, an average error performance was computed within trials of each condition (i.e., 16, eight for each spatial frame) for each patient. The average egocentric and allocentric performances of the patient that could not complete the “immersive” condition were imputed with a random forest algorithm using *mice* package (van Buuren and Groothuis-Oudshoorn, 2011). No trial was removed in all the analyses. These averaged performances in the six patients were used to carry out the analyses as described in the *Singcar* package. The level of significance was set to alpha = 0.05.

## Results

Table 1 shows Crawford and colleagues' *t*-tests (1998) for demographics, neuropsychological, and clinical characteristics of the participants.

### Neuropsychological and psychosis tests

None of the patients included in the study had an equivalent score (Zucchella et al., 2018) of zero in the ACE-R, Corsi block-tapping test (Monaco et al., 2012), and Corsi supra-span test (Spinnler and Tognoni, 1987). That is to say that global cognition, short-term and long-term visuospatial memory were not below 2 SD of the normative population mean. In addition, short-term and long-term visuospatial memory performance was similar between A.A. and the control group. Regarding ACE-R sub-scores (attention-orientation, memory, language, fluency, visuospatial functioning) any differences were found between A.A. and the PD control group. Lastly, A.A. presented mild depressive symptoms (i.e., Geriatric Depression Scale > 5; Laudisio et al., 2018) but this was not significantly different from the control group. See Table 1 for comparisons between A.A. and the control group.

We administered psychosis scales only to A.A. because the control group did not report symptoms of psychosis and hallucinations. We use the normative data of the scales to assess A.A. profile. As shown in Table 2, A.A. had greater perceptual/hallucinations problems compared to the Italian normative data of the non-motor symptoms scale (Cova et al., 2017) (*z*-score of 1.67). In addition, compared to PD patients with psychosis included in the validation of the psychosis scale (PS) by Ondo et al. (2015), A.A. showed a greater frequency of visual hallucinations (*z*-score of 0.89) and sense of presence frequency like the other PD psychotic patients (*z*-score of −0.04). Delusion was not reported by the patient (*z*-score of −0.69), and insight is more preserved than in other PD patients with psychotic symptoms (*z*-score of −1.06). In general, his symptoms are slightly (*z*-score of −0.67) less severe than other PD patients with psychosis.

### Global egocentric and allocentric task performance

We investigated egocentric and allocentric dissociation, regardless of the navigation interfaces, in A.A. using the criteria by McIntosh (2018) and the RSDT (Crawford and Garthwaite, 2005). We found a significant between-task classical dissociation in A.A. compared to the control group (*t*_4_ = 4.52, *p* = 0.011, *Z*_DCC_ = 8.49).

A.A. showed greater error in virtual meters (less accuracy) during the egocentric (*M* = 25.17; standardized score = 1.69) compared to the allocentric (*M* = 19.69; standardized score = 0.05) frame recall condition. Conversely in the control group performance was similar for the egocentric (*M* = 20.37, SD = 2.85) and allocentric (*M* = 19.63, SD = 1.35) frame recall condition. This result is confirmed by the strong dissociation effect size (*Z*_DCC_ = 8.49) of the RSDT. This value is well over three standard deviations from the mean difference in the control group (the mean difference in controls is zero). In addition, the estimated proportion of the control population that would exhibit a difference score between the two measures above A.A. is very low (0.53%). See Supplementary Figure 1 for individual trends for gender and motor asymmetry onset.

**Figure 1:**
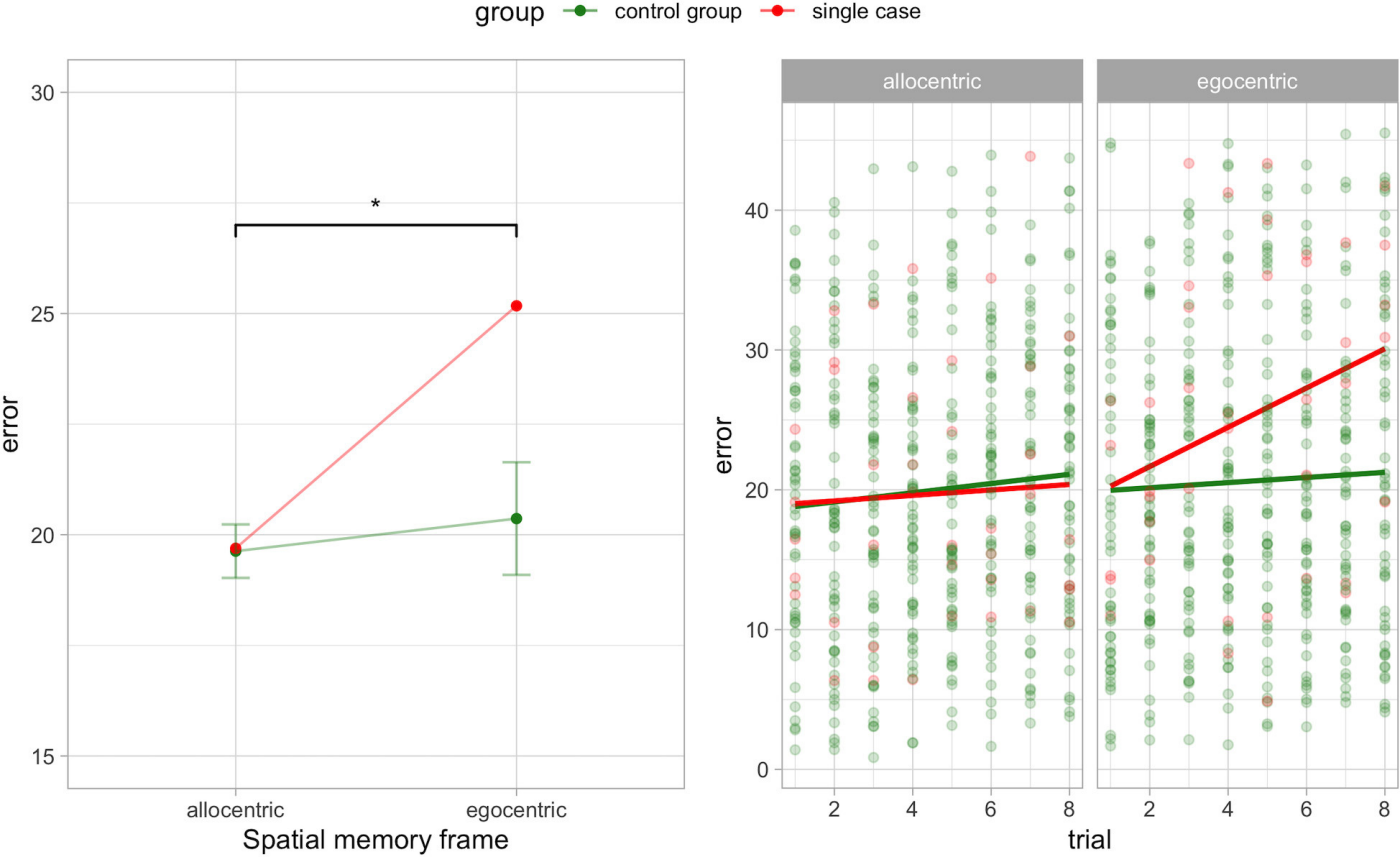
Egocentric-allocentric dissociation **(left panel)** and testing effect **(right panel)** for the egocentric and allocentric frame in A.A. and the control group (PD without hallucinations). Mean and 95% CI are shown for the control group in the left panel. ^*^p < 0.05.

In addition, we analyzed the association between trials and error to evaluate any source of testing effect. We found a statistical tendency for egocentric spatial frame in the case report (ρ = 0.3, *p* = 0.061). This shows a positive association in A.A. between error during egocentric recall and testing trials. The other associations in the control group and A.A. were absent and not significant. See Figure 1 for the classical dissociation between egocentric and allocentric task performance.

### Role of bodily cues on egocentric and allocentric performance

In addition, we studied the impact of egocentric and allocentric spatial memory accuracy depending on the involvement of bodily cues (i.e., motor commands, proprioception, vestibular) recruited during the immersive navigation compared to the passive navigation (i.e., visual information only). We found that providing encoding and retrieval with bodily information cancels the dissociation between the egocentric and allocentric memory performance in A.A. compared to the control group (*t*_4_ = 0.34, *p* = 0.748, *Z*_DCC_ = −0.44).

**Figure 2:**
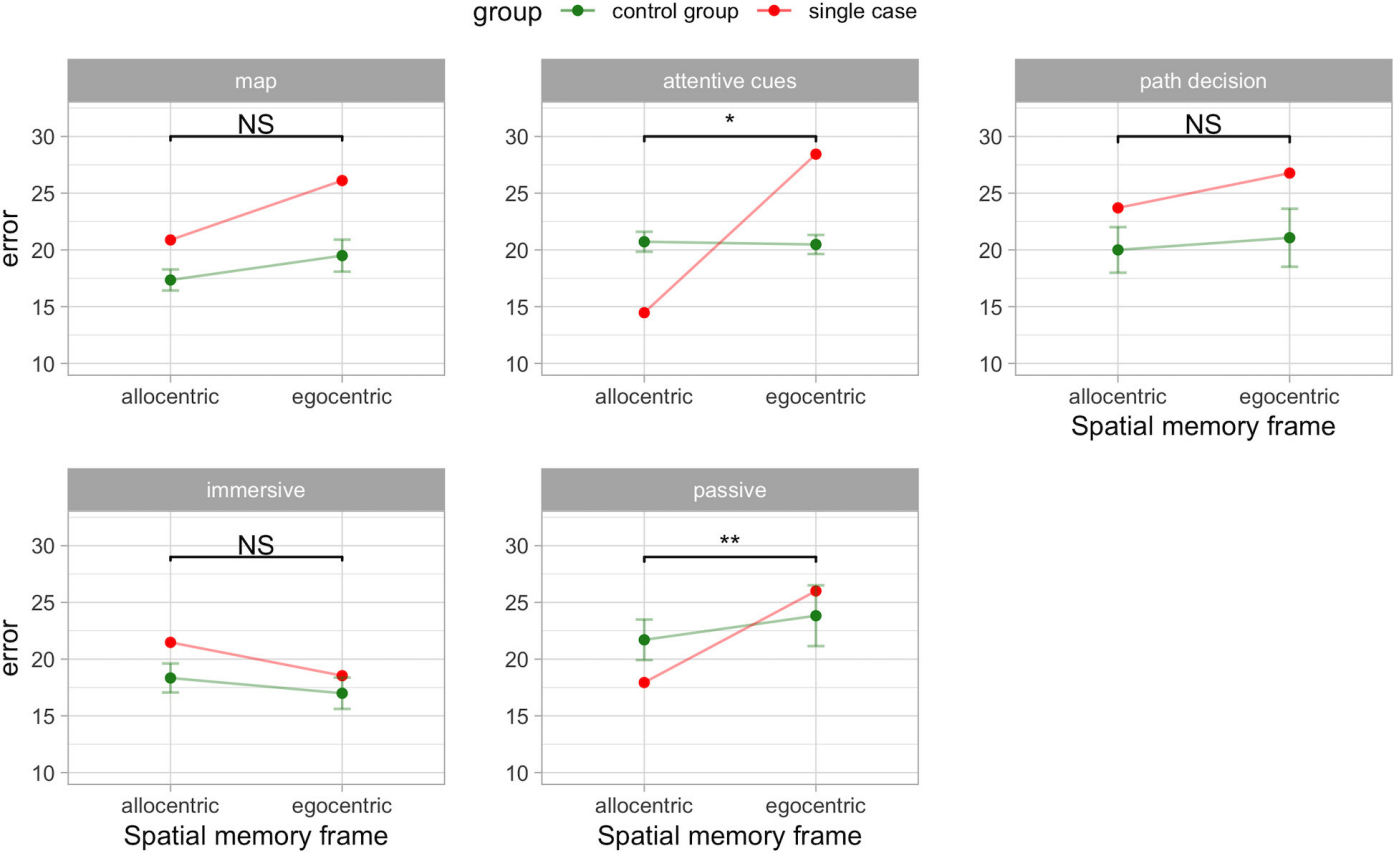
Egocentric-allocentric dissociation in A.A. compared to the control group (PD without hallucinations) in the VR navigation conditions. Mean and 95% CI are shown for the control group. ^*^p < 0.05; ^**^p < 0.01; NS: not significant.

The passive navigation interface significantly affected the egocentric and allocentric spatial memory performance (*t*_4_ = 4.66, *p* = 0.001, *Z*_DCC_ = 8.87). A.A. showed greater error in virtual meters (less accuracy) during the egocentric (*M* = 26.01; standardized score = 0.36) compared to the allocentric (*M* = 17.94; standardized score = −0.94) frame recall condition. Conversely in the control group performance was similar for the egocentric (*M* = 24.83, SD = 5.99) and allocentric (*M* = 21.7, SD = 4) frame recall condition. This result is confirmed by the strong dissociation effect size (*Z*_DCC_ = 8.87) of the RSDT. This value is well over three standard deviations from the mean difference in the control group (the mean difference in controls is zero). In addition, the estimated proportion of the control population that would exhibit a difference score between the two measures above A.A. is very low (0.48%). Figure 2 shows the results in the “immersive” and “passive” conditions.

### Role of cognitive cues on egocentric and allocentric performance

Lastly, we studied the impact of egocentric and allocentric spatial memory accuracy depending on the involvement of cognitive cues provided by the “map,” “path decision,” and “attentive cues” interfaces. We found that providing encoding with an interactive map did not result in the classical dissociation between egocentric and allocentric measures (*t*_4_ = 0.48, *p* = 0. 655, *Z*_DCC_ = 0.68). Similarly, this happened also in the “path decision” condition where the participants could freely move during encoding (*t*_4_ = 0.18, *p* = 0. 864, *Z*_DCC_ = 0.25).

Interestingly, we found a classical dissociation between the egocentric and allocentric performance in the “attentive cues” condition (*t*_4_ = 3.89, *p* = 0. 018, *Z*_DCC_ = 5.40). A.A. showed greater error in virtual meters (less accuracy) during the egocentric (*M* = 28.44; standardized score = 4.24) compared to the allocentric (*M* = 14.47; standardized score = −3.18) frame recall condition. Conversely in the control group performance was similar for the egocentric (*M* = 20.48, SD = 1.88) and allocentric (*M* = 20.72, SD = 1.97) frame recall condition. This result is confirmed by the strong dissociation effect size (*Z*_DCC_ = 5.40) of the RSDT. This value is well over three standard deviations from the mean difference in the control group (the mean difference in controls is zero). In addition, the estimated proportion of the control population that would exhibit a difference score between the two measures above A.A. is very low (0.89%). Figure 2 shows the impact of the cognitive cues on the patients.

## Discussion

In this study, we sought to explore the dissociation between egocentric and allocentric spatial memory under different navigation conditions in a patient with PD and visual hallucinations. We found a classical dissociation, where a greater deficit in the egocentric than the allocentric frame was found in A.A. compared to the control group. This is also confirmed by an increasing error in the consecutive testing trials for the egocentric but not allocentric spatial memory frame. In addition, we found a different influence of the navigation interface on spatial memory frame. Particularly, “passive” and “attentional cues” interfaces significantly affected the egocentric spatial memory in the case report. For the “immersive” one, the absence of significant classical dissociation and better egocentric than allocentric performance could hint that immersive VR provides beneficial bodily cues, particularly for the egocentric frame. These findings could suggest that visual hallucinations in PD are related to an altered egocentric spatial frame of reference and that bodily and cognitive cues could be crucial aspects to consider when studying and treating egocentric processing and hallucinations in PD.

We extended previous research that showed that spatial cognition is affected in PD with visual hallucinations (Koerts et al., 2010; Barnes and Boubert, 2011). We found that egocentric processing is more impaired than the allocentric frame of reference. This is in line with theories and findings that hint that the integration of bodily information into a coherent egocentric frame of reference is crucial in PD illusions and hallucinations (Corlett et al., 2019; Bernasconi et al., 2021).

Interestingly, bodily cues provided by immersive VR improve egocentric processing in the case report and ameliorate this performance also in the control group. This is in line with previous research that shows how immersive VR can be a powerful tool to provide body-based information during spatial navigation (Taube et al., 2013; Tuena et al., 2021b). However, the impact of body-based information in allocentric processing is still a matter of debate (Huffman and Ekstrom, 2019, 2021; Steel et al., 2021) and is thought to be independent of bodily information and mainly supported by the visual system (Chen et al., 2013). This might explain why, during the passive condition, we found the greatest impact on the allocentric rather than on the egocentric performance. It is possible that passively watching the navigation on the PC screen, enabled A.A. to better focus on the visual elements presented in the environment and this increased allocentric memory. Contrarily, in the control group the passive navigation had a detrimental effect on both spatial frames of reference (Tuena et al., 2019). This may hint that visual processing of the space in patients with hallucinations could be improved through techniques that enhance space and attention processing (Koerts et al., 2010).

Regarding the cognitive cues, our results are in line with Cogné et al. (2018). They showed that in mild cognitive impairment the map is not useful in this population. In contrast to previous research on aging, we found that free route-decision do not improve spatial memory (Jebara et al., 2014). Route decision-making relies on executive functions (Viard et al., 2011), PD patients with hallucinations were found to have a greater deficit in this domain compared to PD individuals without hallucinations (Barnes and Boubert, 2008). It is possible that overloading frontal functioning in the condition without directional cues (i.e., line to follow) has a detrimental effect on spatial memory in PD patients that also have hallucinations. Furthermore, we found that in the “attentive cues” condition A.A. had the best allocentric performance compared to all the other interfaces, which is in line with a research study that showed that spatial attention deployment is linked to a preference to use an allocentric strategy (Lithfous et al., 2014; Drisdelle et al., 2017). Indeed, PD patients with visual hallucinations were found to have deficits in the domain of space perception and sustained attention compared to a control PD group (Koerts et al., 2010). It is possible that the use of attentional spatial markers improved the allocation of attention to salient elements of the environment that enhanced allocentric memory. Attentional resources seem to affect mainly egocentric processing and this is in line with the theories that suggest a critical involvement of attention in hallucination in PD (Collerton et al., 2005; Onofrj et al., 2013). However, this set of conclusions on the role of bodily and cognitive cues needs further evidence from studies on PD.

It is possible to hypothesize a potential link among the right striatum, egocentric deficit, and visual hallucinations/illusions. Typically, the nigrostriatal system is affected asymmetrically and leads to core motor signs contralateral to the degenerated regions of the basal ganglia (Djaldetti et al., 2006). Some studies showed that patients who developed hallucinations had at baseline more impairment in DaT in the right ventral striatum (Jaakkola et al., 2017) and right caudate uptake (Kiferle et al., 2014). This is in line with A.A. DaT scan carried out in 2019 (see “Participants” section). It is possible that the egocentric frame, due to its sensorimotor and body-based nature and its neural basis (i.e., right striatum; Doeller et al., 2008), is an impaired cognitive mechanism that could play a role in PD visual hallucinations.

This study has some limitations that should be mentioned. This is an exploratory study with a case report to test a preliminary set of conclusions, larger sample size groups are preferred. Then, more advanced research paradigms could balance patients depending on striatal/motor asymmetry. Future studies could focus on the use of innovative technologies to study or rehabilitate this condition (see also the concept of “embodied medicine” and “digital biomarkers” Riva et al., 2018, 2019; Di Lernia et al., 2019, 2020).

Understanding the neurocognitive underpinnings of visual hallucinations is crucial for determining their causes and potential treatments. VR navigation tasks could provide useful behavioral data on the cognitive mechanisms that contribute to hallucinations in PD. Lastly, VR could be used to design non-pharmacological interventions to reduce hallucinations by improving egocentric processing.

## Data availability statement

The datasets presented in this article are not readily available because. This is a case report and data cannot be disclosed. Requests to access the datasets should be directed at c.tuena@auxologico.

## Ethics statement

The studies involving human participants were reviewed and approved by IRCCS Istituto Auxologico Italiano. The patients/participants provided their written informed consent to participate in this study. Written informed consent was obtained from the individual(s) for the publication of any potentially identifiable images or data included in this article.

## Author contributions

Conceptualization, writing, experimental examination, statistical analyses, and funding acquisition: CT. Clinical examination: LC, EP, KG, IM, and CT. Supervision: GR, MS-B, and EP. All authors have read and agreed to the published version of the manuscript.

## Funding

Research funded by the Italian Ministry of Health (SG-2018-12368175) and partially supported by Ricerca Corrente (EgoHalPD).

## Conflict of interest

The authors declare that the research was conducted in the absence of any commercial or financial relationships that could be construed as a potential conflict of interest.

## Publisher's note

All claims expressed in this article are solely those of the authors and do not necessarily represent those of their affiliated organizations, or those of the publisher, the editors and the reviewers. Any product that may be evaluated in this article, or claim that may be made by its manufacturer, is not guaranteed or endorsed by the publisher.

## References

[B1] Albani G. Pedroli E. Cipresso P. Bulla D. Cimolin V. Thomas A.. (2015). Visual hallucinations as incidental negative effects of virtual reality on Parkinson's disease patients: a link with neurodegeneration? Parkinsons Dis. 2015:194629. 10.1155/2015/194629 26064775 PMC4441992

[B2] Arnulf I. Bonnet A. M. Damier P. Bejjani B. P. Seilhean D. Derenne J. P.. (2000). Hallucinations, REM sleep, and Parkinson's disease: a medical hypothesis. Neurology 55, 281–288. 10.1212/WNL.55.2.281 10908906

[B3] Arzy S. Seeck M. Ortigue S. Spinelli L. Blanke O. (2006). Induction of an illusory shadow person. Nature 443, 287–287. 10.1038/443287a 16988702

[B4] Barnes J. Boubert L. (2008). Executive functions are impaired in patients with Parkinson's disease with visual hallucinations. J. Neurol. Neurosurg. Psychiatry 79, 190–192. 10.1136/jnnp.2007.116202 18202206

[B5] Barnes J. Boubert L. (2011). Visual memory errors in Parkinson's disease patient with visual hallucinations. Int. J. Neurosci. 121, 159–164. 10.3109/00207454.2010.539308 21138396

[B6] Barnes J. Boubert L. Harris J. Lee A. David A. S. (2003). Reality monitoring and visual hallucinations in Parkinson's disease. Neuropsychologia 41, 565–574. 10.1016/S0028-3932(02)00182-3 12559149

[B7] Berardelli A. Wenning G. K. Antonini A. Berg D. Bloem B. R. Bonifati V.. (2013). EFNS/MDS-ES recommendations for the diagnosis of Parkinson's disease. Eur. J. Neurol. 20, 16–34. 10.1111/ene.12022 23279440

[B8] Bernasconi F. Blondiaux E. Potheegadoo J. Stripeikyte G. Pagonabarraga J. Bejr-Kasem H.. (2021). Robot-induced hallucinations in Parkinson's disease depend on altered sensorimotor processing in fronto-temporal network. Sci. Transl. Med. 13, eabc8362. 10.1126/scitranslmed.abc8362 33910980

[B9] Bertram K. Williams D. R. (2012). Visual hallucinations in the differential diagnosis of parkinsonism. J. Neurol. Neurosurg. Psychiatry 83, 448–452. 10.1136/jnnp-2011-300980 22228724 PMC3297805

[B10] Buckner R. L. Andrews-Hanna J. R. Schacter D. L. (2008). The brain's default network: anatomy, function, and relevance to disease. Ann. N. Y. Acad. Sci. 1124, 1–38. 10.1196/annals.1440.011 18400922

[B11] Buckner R. L. Carroll D. C. (2007). Self-projection and the brain. Trends Cogn. Sci. 11, 49–57. 10.1016/j.tics.2006.11.004 17188554

[B12] Burgess N. (2008). Spatial cognition and the brain. Ann. N. Y. Acad. Sci. 1124, 77–97. 10.1196/annals.1440.002 18400925

[B13] Callesen M. B. Hansen K. V. Gjedde A. Linnet J. Møller A. (2013). Dopaminergic and clinical correlates of pathological gambling in Parkinson's disease: a case report. Front. Behav. Neurosci. 7:95. 10.3389/fnbeh.2013.00095 23908610 PMC3725950

[B14] Chen G. King J. A. Burgess N. O'Keefe J. (2013). How vision and movement combine in the hippocampal place code. Proc. Natl. Acad. Sci. USA. 110, 378–383. 10.1073/pnas.1215834110 23256159 PMC3538268

[B15] Chersi F. Burgess N. (2015). The cognitive architecture of spatial navigation: hippocampal and striatal contributions. Neuron 88, 64–77. 10.1016/j.neuron.2015.09.021 26447573

[B16] Chrastil E. R. Warren W. H. (2012). Active and passive contributions to spatial learning. Psychon. Bull. Rev. 19, 1–23. 10.3758/s13423-011-0182-x 22083627

[B17] Cogné M. Auriacombe S. Vasa L. Tison F. Klinger E. Sauzéon H.. (2018). Are visual cues helpful for virtual spatial navigation and spatial memory in patients with mild cognitive impairment or Alzheimer's disease? Neuropsychology 32, 385–400. 10.1037/neu0000435 29809030

[B18] Collerton D. Perry E. McKeith I. (2005). Why people see things that are not there: a novel perception and attention deficit model for recurrent complex visual hallucinations. Behav. Brain Sci. 28, 737–757. 10.1017/S0140525X05000130 16372931

[B19] Corlett P. R. Horga G. Fletcher P. C. Alderson-Day B. Schmack K. Powers A. R.. (2019). Hallucinations and strong priors. Trends Cogn. Sci. 23, 114–127. 10.1016/j.tics.2018.12.001 30583945 PMC6368358

[B20] Cova I. Battista D. i. Vanacore M. E. Papi N. Alampi C. P. Rubino G.. (2017). Validation of the Italian version of the non motor symptoms scale for Parkinson's disease. Parkinsonism Relat. Disord. 34, 38–42. 10.1016/j.parkreldis.2016.10.020 28029554

[B21] Crawford J. R. Garthwaite P. H. (2005). Testing for suspected impairments and dissociations in single-case studies in neuropsychology: evaluation of alternatives using monte carlo simulations and revised tests for dissociations. Neuropsychology 19, 318–331. 10.1037/0894-4105.19.3.318 15910118

[B22] Crawford J. R. Garthwaite P. H. Porter S. (2010). Point and interval estimates of effect sizes for the case-controls design in neuropsychology: rationale, methods, implementations, and proposed reporting standards. Cogn. Neuropsychol. 27, 245–260. 10.1080/02643294.2010.513967 20936548

[B23] Crawford J. R. Howell D. C. Garthwaite P. H. (1998). Payne and Jones revisited: estimating the abnormality of test score differences using a modified paired samples t test. J. Clin. Exp. Neuropsychol. 20, 898–905. 10.1076/jcen.20.6.898.1112 10484700

[B24] Di Lernia D. Lacerenza M. Ainley V. Riva G. (2020). Altered interoceptive perception and the effects of interoceptive analgesia in musculoskeletal, primary, and neuropathic chronic pain conditions. J. Pers. Med. 10, 201. 10.3390/jpm10040201 33138185 PMC7712753

[B25] Di Lernia D. Serino S. Polli N. Cacciatore C. Persani L. Riva G. (2019). Interoceptive axes dissociation in anorexia nervosa: A single case study with follow up post-recovery assessment. Front. Psychol. 9, 2488. 10.3389/fpsyg.2018.02488 30705649 PMC6345152

[B26] Diederich N. J. Fénelon G. Stebbins G. Goetz C. G. (2009). Hallucinations in Parkinson disease. Na. Rev. Neurol. 5, 331–342. 10.1038/nrneurol.2009.62 19498436

[B27] Diederich N. J. Goetz C. G. Stebbins G. T. (2005). Repeated visual hallucinations in Parkinson's disease as disturbed external/internal perceptions: focused review and a new integrative model. Mov. Disord. 20, 130–140. 10.1002/mds.20308 15486924

[B28] Djaldetti R. Ziv I. Melamed E. (2006). The mystery of motor asymmetry in Parkinson's disease. Lancet Neurol. 5, 796–802. 10.1016/S1474-4422(06)70549-X 16914408

[B29] Doeller C. F. King A. J. Burgess N. (2008). Parallel striatal and hippocampal systems for landmarks and boundaries in spatial memory. Proc. Natl. Acad. Sci. USA. 105, 5915–5920. 10.1073/pnas.0801489105 18408152 PMC2311337

[B30] Drisdelle B. L. Konishi K. Diarra M. Bohbot V. D. Jolicoeur P. West G. L.. (2017). Electrophysiological evidence for enhanced attentional deployment in spatial learners. Exp. Brain Res. 235, 1387–1395. 10.1007/s00221-017-4884-9 28229169

[B31] Fernandez-Baizan C. Garcia M. P. F. DIaz-Caceres E. Menendez-Gonzalez M. Arias J. L. Mendez M. (2020). Patients with Parkinson's disease show alteration in their visuospatial abilities and in their egocentric and allocentric spatial orientation measured by card placing tests. J. Parkinsons Dis. 10, 1807–1816. 10.3233/JPD-202122 33016894

[B32] Ffytche D. H. Creese B. Politis M. Chaudhuri K. R. Weintraub D. Ballard C.. (2017). The psychosis spectrum in Parkinson disease. Nat. Rev. Neurol. 13, 81–95. 10.1038/nrneurol.2016.200 28106066 PMC5656278

[B33] Franciotti R. Falasca N. W. Bonanni L. Anzellotti F. Maruotti V. Comani S.. (2013). Default network is not hypoactive in dementia with fluctuating cognition: an Alzheimer disease/dementia with lewy bodies comparison. Neurobiol. Aging 34, 1148–1158. 10.1016/j.neurobiolaging.2012.09.015 23063646

[B34] Glass P. G. Lees A. J. Bacellar A. Zijlmans J. Katzenschlager R. Silveira-Moriyama L.. (2012). The clinical features of pathologically confirmed vascular Parkinsonism. J. Neurol. Neurosurg. Psychiatry 83, 1027–1029. 10.1136/jnnp-2012-302828 22960987

[B35] Goldman J. G. Holden S. Bernard B. Ouyang B. Goetz C. G.. (2013). Defining optimal cutoff scores for cognitive impairment using movement disorder society task force criteria for mild cognitive impairment in Parkinson's disease. Mov. Disord. 28, 1972–1979. 10.1002/mds.25655 24123267 PMC4164432

[B36] Guderian S. Dzieciol X. A. M. Gadian D. G. Jentschke X. S. Doeller C. F. Burgess N.. (2015). Hippocampal volume reduction in humans predicts impaired allocentric spatial memory in virtual-reality navigation. J. Neurosci. 35, 14123–14131. 10.1523/JNEUROSCI.0801-15.2015 26490854 PMC4683681

[B37] Hartevelt T. J. V. Cabral J. Møller A. FitzGerald J. J. Green A. L. Aziz T. Z.. (2015). Evidence from a rare case study for hebbian-like changes in structural connectivity induced by long-term deep brain stimulation. Front. Behav. Neurosci. 9, 167. 10.3389/fnbeh.2015.00167 26175675 PMC4485173

[B38] Huffman D. J. Ekstrom A. D. (2019). A modality-independent network underlies the retrieval of large-scale spatial environments in the human brain. Neuron 104, 611–622.e7. 10.1016/j.neuron.2019.08.012 31540825 PMC6842116

[B39] Huffman D. J. Ekstrom A. D. (2021). An important step toward understanding the role of body-based cues on human spatial memory for large-scale environments. J. Cogn. Neurosci. 33, 167–179. 10.1162/jocn_a_01653 33226317

[B40] Humphries S. Holler J. Crawford T. J. Herrera E. Poliakoff E. (2016). A third-person perspective on co-speech action gestures in Parkinson's disease. Cortex 78, 44–54. 10.1016/j.cortex.2016.02.009 26995225 PMC4865523

[B41] Jaakkola E. Joutsa J. Mäkinen E. Johansson J. Kaasinen V. (2017). Ventral striatal dopaminergic defect is associated with hallucinations in Parkinson's disease. Eur. J. Neurol. 24, 1341–1347. 10.1111/ene.13390 28834102

[B42] Jebara N. Orriols E. Zaoui M. Berthoz A. Piolino P. (2014). Effects of enactment in episodic memory: a pilot virtual reality study with young and elderly adults. Front. Aging Neurosci. 6, 338. 10.3389/fnagi.2014.00338 25566069 PMC4269133

[B43] Kiferle L. Ceravolo R. Giuntini M. Linsalata G. Puccini G. Volterrani D.. (2014). Caudate dopaminergic denervation and visual hallucinations: evidence from a 123I-FP-CIT SPECT study. Parkinsonism Relat. Disord. 20, 761–765. 10.1016/j.parkreldis.2014.04.006 24787757

[B44] Koerts J. Borg M. A. J. P. Meppelink A. M. Leenders K. L. T. (2010). Attentional and perceptual impairments in Parkinson's disease with visual hallucinations. Parkinsonism Relat. Disord. 16, 270–274. 10.1016/j.parkreldis.2010.01.003 20153970

[B45] Kuehn E. Perez-Lopez M. B. Diersch N. Döhler J. Wolbers T. Riemer M.. (2017). Embodiment in the aging mind. Neurosci. Biobehav. Rev. 86, 207–225. 10.1016/j.neubiorev.2017.11.016 29175306

[B46] Laudisio A. Antonelli Incalzi R. Gemma A. Marzetti E. Pozzi G. Padua L.. (2018). Definition of a geriatric depression scale cutoff based upon quality of life: a population-based study. Int. J. Geriatr. Psychiatry 33, e58–e64. 10.1002/gps.4715 28370551

[B47] Lithfous S. Dufour A. Blanc F. Després O. (2014). Allocentric but not egocentric orientation is impaired during normal aging: an ERP study. Neuropsychology 28, 761–771. 10.1037/neu0000084 24749730

[B48] Litvan I. J. G. Goldman J. G. Tröster A. I. Schmand B. A. Weintraub D. Petersen R. C.. (2012). Diagnostic criteria for mild cognitive impairment in Parkinson's disease: movement disorder society task force guidelines. Mov. Disord. 27, 349–356. 10.1002/mds.24893 22275317 PMC3641655

[B49] McIntosh R. D. (2018). Simple dissociations for a higher-powered neuropsychology. Cortex 103, 256–265. 10.1016/j.cortex.2018.03.015 29673782

[B50] Monaco M. Costa A. Caltagirone C. Carlesimo G. A. (2012). Forward and backward span for verbal and visuo-spatial data : standardization and normative data from an Italian adult population. Neurol. Sci. 34, 749–754. 10.1007/s10072-012-1130-x 22689311

[B51] Muller A. J. Shine J. M. Halliday G. M. Lewis S. J. G. (2014). Visual hallucinations in Parkinson's disease: theoretical models. Mov. Disord. 29, 1591–1598. 10.1002/mds.26004 25154807

[B52] Ondo W. G. Sarfaraz S. Lee M. J. (2015). A novel scale to assess psychosis in patients with Parkinson's disease. J. Clin. Mov. Disord. 2, 1–5. 10.1186/s40734-015-0024-5 26788353 PMC4711049

[B53] Onofrj M. Taylor J. P. Monaco D. Franciotti R. Anzellotti F. Bonanni L.. (2013). Visual hallucinations in PD and Lewy body dementias: old and new hypotheses. Behav. Neurol. 27, 479–493. 10.1155/2013/703924 23242366 PMC5215646

[B54] Pigliautile M. Chiesi F. Rossetti S. Conestabile della Staffa M. Ricci M. Federici S.. (2015). Normative data for the ACE-R in an Italian population sample. Neurol. Sci. 36, 2185–2190. 10.1007/s10072-015-2330-y 26216492

[B55] R Core Team (2013). R: A Language and Environment for Statistical Computing. Wien: R Foundation for Statistical Computing. Available online at: https://www.r-project.org/

[B56] Riley D. S. Barber M. S. Kienle G. S. Aronson J. K. von Schoen-Angerer T. Tugwell P.. (2017). CARE guidelines for case reports: explanation and elaboration document. J. Clin. Epidemiol. 89, 218–235. 10.1016/j.jclinepi.2017.04.026 28529185

[B57] Riva G. (2018). The neuroscience of body memory: from the self through the space to the others. Cortex 104, 241–260. 10.1016/j.cortex.2017.07.013 28826604

[B58] Riva G. Wiederhold B. K. Chirico A. Di Lernia D. Mantovani F. Gaggioli A. (2018). Brain and virtual reality: What do they have in common and how to exploit their potential. Ann. Rev. Cyberther. Telemed. 16, 3–7.

[B59] Riva G. Wiederhold B. K. Di Lernia D. Chirico A. Riva E. F. M. Mantovani F.. (2019). Virtual reality meets artificial intelligence: The emergence of advanced digital therapeutics and digital biomarkers. Ann. Rev. Cyberther. Telemed. 17, 3–7.

[B60] Spinnler H. Tognoni G. (1987). Standardizzazione e taratura italiana di test neuropsicologici. Ital. J. Neurol. Sci. 8 (Suppl): 1–120. 3330072

[B61] Steel A. Robertson C. E. Taube J. S. (2021). Current promises and limitations of combined virtual reality and functional magnetic resonance imaging research in humans: A commentary on huffman and ekstrom (2019). J. Cogn. Neurosci. 33, 159–166. 10.1162/jocn_a_01635 33054553 PMC10284033

[B62] Taube J. S. Valerio S. Yoder R. M. (2013). Is navigation in virtual reality with fMRI really navigation? J. Cogn. Neurosci. 25, 1008–1019. 10.1162/jocn_a_00386 23489142 PMC10271117

[B63] Thurm F. Schuck N. W. Fauser M. Doeller C. F. Stankevich Y. Evens R.. (2016). Dopamine modulation of spatial navigation memory in Parkinson's disease. Neurobiol. Aging 38, 93–103. 10.1016/j.neurobiolaging.2015.10.019 26827647

[B64] Tuena C. Mancuso V. Stramba-Badiale C. Pedroli E. Stramba-Badiale M. Riva G.. (2021a). Egocentric and allocentric spatial memory in mild cognitive impairment with real-world and virtual navigation tasks: a systematic review. J. Alzheimers Dis. 79, 95–116. 10.3233/JAD-201017 33216034 PMC7902987

[B65] Tuena C. Serino S. Dutriaux L. Riva G. Piolino P. (2019). Virtual enactment effect on memory in young and aged populations : a systematic review. J. Clin. Med. 8, 620. 10.3390/jcm8050620 31067784 PMC6572276

[B66] Tuena C. Serino S. Pedroli E. Stramba-Badiale M. Riva G. Repetto C. (2021b). Building embodied spaces for spatial memory neurorehabilitation with virtual reality in normal and pathological aging. Brain Sci. 11, 1067. 10.3390/brainsci11081067 34439686 PMC8393878

[B67] van Buuren S. Groothuis-Oudshoorn K. (2011). Mice: multivariate imputation by chained equations in R. J. Stat. Softw. 45, 1–67. 10.18637/jss.v045.i03

[B68] Viard A. Doeller C. F. Hartley T. Bird C. M. Burgess N. (2011). Anterior hippocampus and goal-directed spatial decision making. J. Neurosci. 31, 4613–4621. 10.1523/JNEUROSCI.4640-10.2011 21430161 PMC6622909

[B69] Vizcarra J. A. Lang A. E. Sethi K. D. Espay A. J. (2015). Vascular Parkinsonism: deconstructing a syndrome. Mov. Disord. 30, 886–894. 10.1002/mds.26263 25997420 PMC4478160

[B70] Williams D. R. Lees A. J. (2005). Visual hallucinations in the diagnosis of idiopathic parkinson's disease: a retrospective autopsy study. Lancet Neurol. 4, 605–610. 10.1016/S1474-4422(05)70146-0 16168928

[B71] Zucchella C. Federico A. Martini A. Tinazzi M. Bartolo M. Tamburin S.. (2018). Neuropsychological testing. Pract. Neurol. 18, 227–237. 10.1136/practneurol-2017-001743 29472384

