# Supplementary material for: Contribution of cognitive and bodily navigation cues to egocentric and allocentric spatial memory in hallucinations due to Parkinson's disease: A case report
Source: Front Behav Neurosci. 2022 Oct 13;16:992498. doi: 10.3389/fnbeh.2022.992498 (PMC9606325; doi:10.3389/fnbeh.2022.992498)
Supplement: Supplementary file 1 [file Data_Sheet_1.docx]

Supplementary Material

# Supplementary Material 1


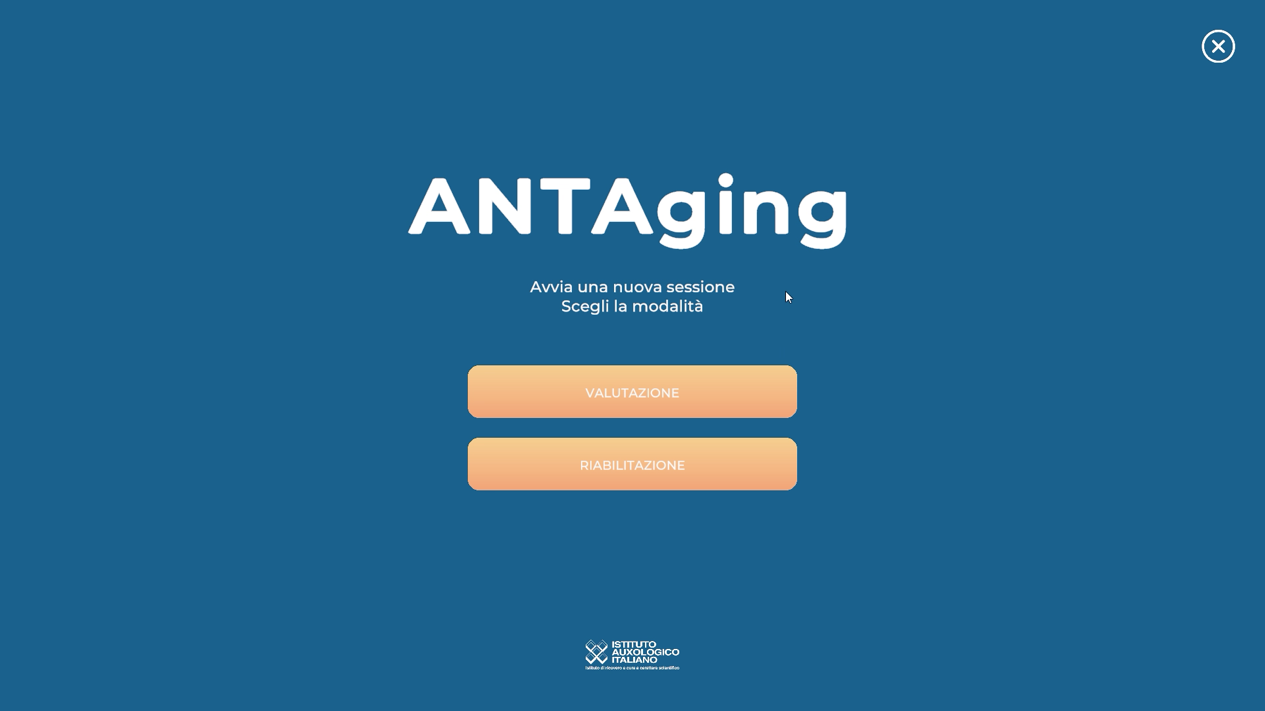


One-object demo of the task. At encoding the navigation cognitive cues are displayed all together for an overview; the retrieval phase consists of only one object relocation.

# Supplementary Figure 1


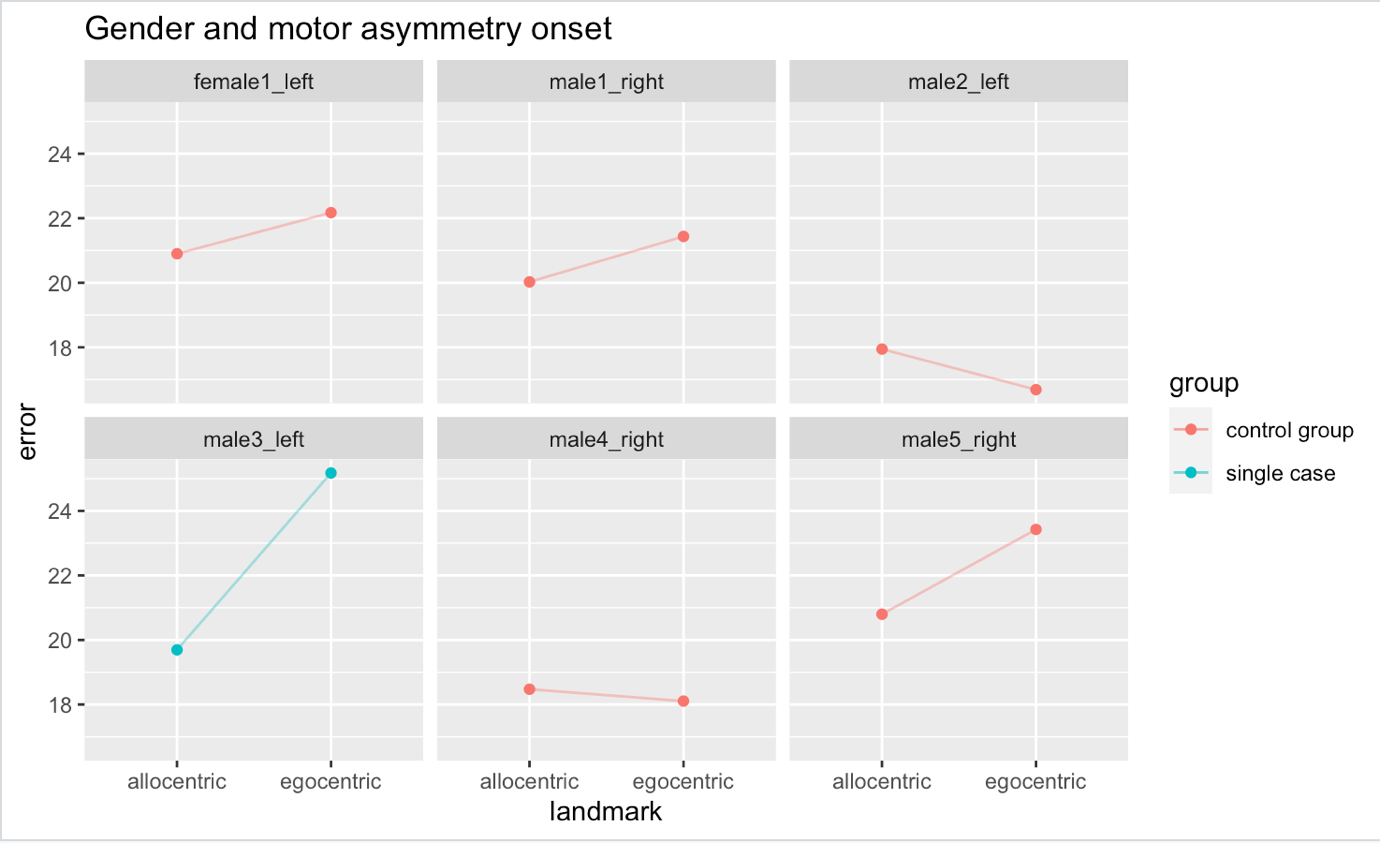


**Supplementary Figure 1.** Egocentric and allocentric spatial memory trends by gender and motor asymmetry.
